# The physiological reaction of Siberian hamsters (*Phodopus sungorus*, Cricetidae) to chemical signals of perspective mating partners before and during courtship

**DOI:** 10.1242/bio.057570

**Published:** 2021-03-26

**Authors:** E. Yu Kondratyuk, P. A. Zadubrovskiy, I. V. Zadubrovskaya, A. V. Sakharov

**Affiliations:** 1 Institute of Systematics and Ecology of Animals, SB RAS, Novosibirsk 630091, Russia; 2 Novosibirsk State Pedagogical University, Novosibirsk 6300126, Russia

**Keywords:** Dwarf hamsters, Immunological and endocrine status, Odor attractiveness, Preference of partner

## Abstract

In this investigation we assessed the physiological reaction of hamsters in response to chemical signals from potential sexual partners, and also after a private meeting with them, which allowed us to ascertain the type of mating system for this species. The reception of olfactory signals led to an increase in peroxidase activity in the blood for both sexes, indicative of activity of a non-specific line of immune defense in recipients. The increase in blood cortisol level in response to the chemical signals of a partner was only observed in females. Males spent more time near samples of estrous females, with elevated levels of cortisol in the urine. In olfactory tests, an hour after grouping all the individuals in pairs there was a significant increase in blood peroxidase activity, which indicates the reaction of a non-specific link in the immune system of partners. This increase was greater in the pairs with a mutual preference. Females from these pairs demonstrated a substantial decrease in stress hormone levels in the plasma after an hour of mating in comparison to females prior to mating, and in non-preferred coupling.

## Abstract (precis)

**Summary:** Perception of olfactory signals before and during courtship lead to an increase of non-specific immune activity. For pairs formed by mutual preference there was a noted decrease of stress-hormone only in females.

## INTRODUCTION

As is well known, social odors play a substantial and often essential role in mammalian reproduction. For example, chemosensory signals can accelerate or decelerate maturation and function in adults of the hypothalamic-pituitary-gonadal (HPG) axis of recipients, such that an increase in HPG function occurs in response to the appearance of mating partners (Boehm et al., 2005) and there is a decrease in HPG function in reproductively sub-optimal conditions (stress effects) (Toufexis et al., 2014). Perception of scents from opposite-sex conspecifics initiates behaviors that increase the likelihood of contacting mates: olfactory investigation, scent marking and vocalizations. And even after contact occurs, the initiation of copulatory behavior in both sexes may still depend on chemosensory cues, perhaps because these cues allow a more detailed evaluation of the mating partner (Petrulis, 2013).

Moreover, the sources of the molecules that make up an animal's ‘individual scent’, including its own secretions, reflect all the processes the in neuroimmune-endocrine system, bacteria burden and food. For example, female house mice prefer scents of dominant males to those of subordinates (Mossman and Drickamer, 1996) and meadow voles prefer chemosignals of males fed on high protein diets, a normally deficient and valuable resource (Ferkin et al., 1997). Female mice likewise decrease their preference for chemosignals from males sub-clinically infected with parasites (Kavaliers and Colwell, 1995). In experimental studies on the separation of the links of the immune system, it has been shown that the above effect develops as a result of activation of a specific line of immune response (Moshkin et al., 2001) because an innate immune response was observed in the opposite. For example, it was shown that there was an increase in female attraction for chemical signals from sick male mice, which resulted from an injection of endotoxine (LPS) (Kondratyuk et al., 2004). It can be explained in that temporary activation of innate response and ‘inevitable recovery’ indicates a high quality male immune system. In turn, reception of olfactory signals leads to the activation of the immune neuroendocrine system. Recent research has shown an increase in the quantity of leukocytes in bronchoalveolar lavage in males after perception of the chemosignals of females (Litvinova et al., 2009). This provides for an anticipatory adaptation of male mice to potential risks of respiratory infections (Litvinova et al., 2010).

However, in recent research on hamsters, this was shown in different information loads of the studied excretions (urine, feces, secretions of the mid-abdominal gland) of male hamsters, depending on the type of animal recipient and the season. An increase in testosterone level in the blood of Eversmann's, Roborovski and Chinese hamsters was noted in response to the smell of female urine in spring and summer. The male's cortisol level was shown to increase after receiving chemical signals of females in spring only in the Chinese and Roborovski hamsters (Kropotkina et al., 2016).

It has been shown that female interest is modulated by the spatial pattern of scent-deposition indicative of a territory owner (Gosling and Roberts, 2001). Female mice and Syrian hamsters prefer scents from males they have previously encountered (Hurst and Beynon, 2004; Johnston, 2008), which is more common for species with a monogamous mating system. Female house mice (*Mus musculus*) produce fewer offspring and offspring survival is lower when they mate with non-preferred males (Drickamer et al. 2003), and females mated to preferred males have litter sizes 31% larger than those mated to non-preferred males (Drickamer et al., 2000).

Despite the multilateral study of the influence of olfactory signals on future life partner choice strategy, the question of differences between physiological reactions upon receipt of chemosignals and personal meeting of an individual with preferred and non-preferred partners has still not been fully answered. It was shown previously by our laboratory that preference in mate choice lead to an increase in fertility in a pair (Potapov et al., 1999), also levels of attractiveness of males with different mating systems was noted (Potapov and Evsikov, 2011). But for those investigations dwarf hamsters were not used and previously this opinion about the mating system of this species was contradictory (Wynne-Edwards et al., 1992; Vasilieva et al., 2017).

In our investigation we analyzed the data of physiological reactions after pair forming and were assumed that the physiological response of an individual to cohabitation depended on the type of distribution of individual sites in the wild and the mating system of the species. Following on from the assumption that the nature of the chemical signal is associated with the state of physiological stress systems, in our work we assessed the immune-neuroendocrine response of the hamster (*Phodopus sungorus*, Pallas, 1773) when exposed to the smell of soiled bedding of potential sexual partner, as well as during a direct meeting and joint keeping of animals in dependence on the degree of their preferences.

## RESULTS

### Donor of chemical signal

A significant negative correlation was noted between the time of sniffing donor-soiled bedding by females and the relative concentration of testosterone in the urine of these males (r=−0.43; *n*=35; *P*<0.01), as well as a weak positive correlation with the level of cortisol in urine (r=0.22; *n*=35; *P*=0.05).

In turn, the attractiveness of the soiled bedding of estrous females presented to males was correlated with the relative level of cortisol (r=0.5; *n*=55; *P*<0.01) and protein in urine samples of females (r=0.4; *n*=59; *P*<0.05). The level of stress hormone index in the urine of preferred females was significantly higher than those not preferred by males or that were not chosen (*P*<0.05; Tukey's HSD test).

### Recipient response

**Fig. 1.**
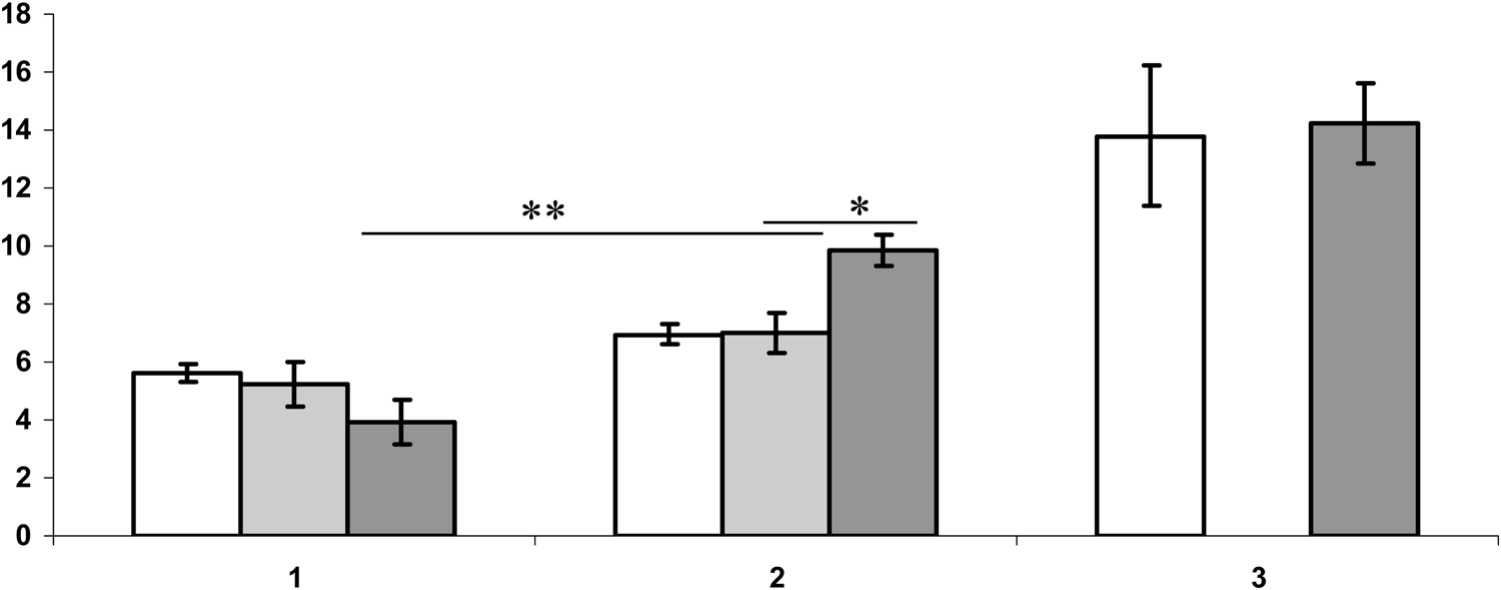
**The peroxidase activity of plasma (a.u.) before (1), and after olfactory test (2) and 1 h after creating pair (3) for males (empty columns), females (non-estrus, light columns; proestrus, dark columns) of dwarf hamsters (*Phodopus sungorus*).** *, *t*=2,6; *P*<0.05; d.f.=62, in comparison with value for stages of cycle **, *t*=4,9; *P*<0.01; d.f.=57, in comparison with female group baseline level (Student's *t*-test).

Based on the results of variance analysis, the endocrine response of females to the scent of a male's soiled bedding was determined by their receptive state (*F*_1;140_=14.2; *P*<0.01). Thus, for non-estrous females, the level of cortisol after perception a chemosignal was higher than at estrous females (*Z*=3.7; d.f.=114; *P*<0.01, U-test). Females spent significantly more time near the samples in a state of pro-estrous (*t*=3.9; *P*<0.01; d.f.=116), in comparison with non-estrous females. The stage of the cycle also determined the level of plasma peroxidase activity after olfactory test, which was significantly higher in estrous females (*t*=2.6; *P*<0.05; d.f.=62) in comparison with non-estrous females (Fig. 1). An increase in peroxidase activity in general is typical for all animals (for females: *t*=2.7; *P*<0.01; d.f.=80; for males: *t*=2.6; *P*<0.01; d.f.=69, in comparison with baseline level; Fig. 1). In males, after reception of female chemosignals, there was no increase in plasma cortisol levels after the tests (*Z*=1.1; d.f.=87; *P* =0.3, U-test, in comparison with the baseline levels).

Analysis of variance, taking into account the factors: decisiveness in the selection and kin of the odor donor, showed the absence of their significant effect on the plasma level of cortisol (*F*_1;167_=0.001; *P*=0.97 and *F*_1;167_=0.1; *P*=0.7, respectively) and testosterone (*F*_1;69_=1.0; *P*=0.3 and *F*_1;69_=0.4; *P*=0.6, respectively). However, in the case of division recipients by sex, a significant effect of the degree of kin on the reaction of males (*F*_1;32_=7.4; *P*=0.01) was noted. For those males there was a significant decrease in the level of cortisol in the blood when receiving signals from sisters (t=2.2; *P*<0.05; d.f.=24; in comparison with non-siblings, preferred females). For females, however, no such dependence was found.

**Fig. 2.**
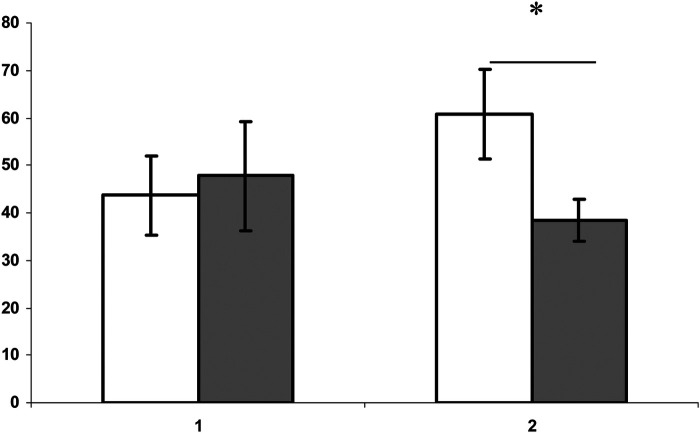
**The concentration of cortisol (ng ml^−1^, axis Y) in plasma after 1 h in pairs: non-preference (empty columns) and mutual preference (dark columns) of dwarf hamsters (*Phodopus sungorus*).** 1, males; 2, females. *, *t*=2.4; *P*<0.05; d.f.=10 (Student's *t*-test).

After identifying preferences in multiple olfactory tests, we formed pairs of females in the pro-estrous stage, selecting non-sibling partners for them. One group consisted of five pairs, all mutually preferring animals. Another group consisted of individuals consistently not preferring each other (five pairs). 1 h after forming pairs, preferred-choice females had a noted tendency to decrease cortisol levels (t=2.4; *P*<0.05; d.f.=10), in comparison with the level of cortisol in the plasma of females from non-preference pairs (Fig. 2).

In general, all animals showed a significant decrease in the level of cortisol after pairing (*F*=91.6; *P*<0.0001; d.f.=16, RM-ANOVA), in comparison to the baseline levels. Plasma peroxidase activity 1 h after pairing was increased in all individuals (*F*=555; d.f.=18; *P*<0.005, RM-ANOVA; in comparison to baseline levels). At the same time, individuals from the mutually preferred pairs had a higher level of peroxidase activity in comparison to the non-preferred (*t*=2.2; d.f.=16; *P*<0.05). After 16 days of keeping hamsters in pairs before separation, we noted a significant decrease in peroxidase activity compared to baseline levels (*F*=1.9; d.f.=18; *P*=0.2, RM-ANOVA), as well as no differences in this indicator between animals of different pairs (*t*=0.2; *P*=0.8; d.f.=9).

## DISCUSSION

The choice of a potential mate in mammals is based on the direct perception of chemical signals containing information that is specific to the species, sex, social and physiology status. Its features, as shown by the recent studies on hamsters – species with clearly well-expressed seasonal changes, are mostly largely determined by the source of signals (type of excreta), as well as by the season of perception/presentation (Feoktistova et al., 2013; Kropotkina et al., 2016). In those studies, females in 30-min tests showed a significant increase in progesterone levels in response to the scent of urine and abdominal gland secretion for conspecific males in the winter and summer. No significant changes in estradiol and cortisol levels were observed in this species in response to exposure to male chemosignals (Kropotkina et al., 2012). It has been shown previously that fecal samples are an additional source of chemosignals for Siberian hamsters in the summer (Novikov, 1988). In our study, we used soiled bedding that consisted of all the excretions of the animal, and this may have caused some of the changes in endocrine reactions previously described for this species. Thus, in response to receiving chemosignals, males were characterized by a decrease in the level of cortisol in plasma after receiving a signal from a sister and an increase upon receiving a signal from unrelated, preferred females, as well as testosterone concentration in response to this stimulus. It is known that the ‘peaks’ of attractiveness in rodents with different mating systems are located in different zones of the aggressiveness scale (Potapov and Evsikov, 2011; Zadubrovskaya, 2011). For the Siberian hamsters in our study, the most preferred mates were low-aggression males, which was confirmed by the obtained physiological parameters: the females paid more attention to samples from individuals in whose urine the concentration of sex steroids was below average values. Also, males with a high level of cortisol on the eve of testing, and occupying a subordinate position or a high hierarchical status in dyadic tests, had a low attractiveness index. A similar picture had already been obtained in studies on the *Lagurus lagurus* by colleagues, who demonstrated that the least attractive males to estrous females were the completely non-aggressive and ‘overly’ aggressive males. At the same time, the attractiveness of these males was negatively correlated with the basic testosterone level, which is consistent with the idea of a monogamous system of mating in this species. It was found that females preferring males with less aggressiveness contribute to the future success of their progeny, because such males have higher potential parental care (Potapova et al., 2016). The preferences of low-aggression individuals were shown not only in monogamous species, but also in the promiscuity species, the narrow-headed vole (Zadubrovskaya, 2011). In our study, the same tendency was revealed, which can be considered as confirmation of the position of facultative monogamy for this species expressed by Wynne-Edwards et al. (1987). In a previous study, a negative correlation of stress-steroid levels and chemo attractiveness in mice was shown (Moshkin et al., 2001), but in our work the opposite situation is noted, with females with a high level of cortisol in urine being preferred.

It is well known that any meeting with an unfamiliar individual or its chemosignal is a stress for an animal (Korobetskiy, 1984), but it may not always be observed in the laboratory, in particular it may be accompanied by an increase in cortisol during the experiment. Although chemosignals from partners in rodents affect the onset of puberty in juveniles and modulate the release of luteotropin by the pituitary gland (Novikov, 1988), a key hormone for activating the common link is related to HPG and HPA axis. In our work, we managed to record an increase in the level of cortisol in the blood of non-estrous females after receiving chemosignals from males, which is generally considered an adaptive mechanism.

Previously, the importance of perceiving odor signals by adult animals via the central nervous system was shown where a significant increase in neural connections in the brains of females after receiving signals from males was observed, which was not the case with samples from castrated animals (Mak et al., 2007).

In our tests it was noted that activation of the innate link of the immune system occurred in both females and males. In male mice, an increase in peroxidase activity in bronchoalveolar lavage fluid was shown after receiving olfactory signals from females (Kontsevaya, 2013). Such evacuation of leukocytes to the place receiving the chemosignal provides greater resistance to experimental respiratory infection (Litvinova et al., 2010). Therefore, chemosignals, in addition to a stimulating effect on sexual function, simultaneously act as intraspecific signals ‘warning’ of an increase in the infectious risk associated with reproduction. Representatives of this genus are characterized by the structure of overlapping habitats, where several individual areas of females are located on the male site. The boundaries of the overlap zones are marked using the secretion of the middle abdominal gland, while odor marks form a network of ‘information signaling points’ actively visited by animals living in the same territory (Wynne-Edwards et al., 1992).

In our study a significant increase of the blood peroxidase activity was noted 1 h after a private meeting with subsequently formed reproductive pairs and the largest increase of the indicator was characteristic of couples seated by mutual preference. At the same time, the females of these pairs showed a decrease in the level of cortisol in the plasma, which corresponded with the results of choosing the preferred partner in the tests. The endocrine reaction of males to females with different degrees of kinship was also noted: an increase in testosterone levels to the smell of unrelated females and a decrease in cortisol levels after receiving signals from related females, which can be explained by the polygynous mating system for this species. Receipt of an odor signal from females by males who made or did not make a choice did not lead to a different increase in the level of the stress hormone, but significantly increased the level of testosterone with equal preference for signals, because sexually mature males of studied species, in particular in the spring, are always ready to meet females. Thus, as a result of our study, we supplemented the information on the basic laws of choice and the physiological response of the body to pairing in individuals of Siberian hamsters: a promiscuous species with elements of optional monogamy, which is mandatory at the time of pairing.

## MATERIALS AND METHODS

### Animals and design

All experimental procedures were performed at ISEA SB RAS (Novosibirsk, Russia). All procedures were performed in accordance with the European Convention for Protection of Vertebrate Animals used for Experimental and other Scientific Purposes. In our experiment we used 30 adult hamsters (19 males, 11 females) without sexual experience at the age of 120–130 days on the beginning of testing. All animals one month after birth in the winter photoperiod (8 L/16D) were placed in individual cages measuring 20×30×25 cm and kept in the spring (12 L/12D) photoperiod, which corresponded to the natural one. The animals had water and food (vegetables, grain, seed and protein) *ad libitum*. The temperature in rooms was maintained at +21±1°С.

### Analysis of behavior

To identify smell preferences a 10-min test was performed with the soiled bedding of the donor. At testing we used the previously calculated social status of males and the degree of family siblings between donors/recipients. The test was carried out in a plastic installation (consisting of nine compartments, 15×15×15 cm each) with fixed time investigation of compartments by animals (by Kondratyuk et al., 2004). A sample was considered preferable if the tested individual spent more than 60% of the common test time. If the difference in the time spent in each of the compartments was less than 60%, the choice was considered as not made. Sample attractiveness index was calculated as the percentage of the total time spent near samples of the donor. A total of 127 olfactory tests were carried out, ten reproductive pairs were formed. Analysis of the time spent by individuals in the compartments made it possible to reveal multiple coincidences (multiple choice of the odor of one sample) of preferences given to a particular partner. In this case, a pair was formed based on mutual preference (Evsikov et al., 2001). In cases of repeated avoidance of the compartment and the coincidence of less attention to the smell of potential partners, the pair was considered formed by mutual non-preference. Pairing was carried out in neutral territory.

### Analysis of metabolites and hormones

To determine the baseline levels of the studied parameters, blood and urine samples were taken 5 days before the start of manipulations. In plasma, the level of peroxidase activity was determined immediately, the rest of the plasma and urine were stored at −20°C until analyte determination. Urine was obtained by depositing an individual into separate cages for half an hour at the same time in the morning, every day, after that it was collected in tubes and frozen. A total of 129 urine samples were collected. The recipient's blood was taken immediately after the end of the 10-min olfactory test. After creating a pair, samples were collected 1 h later, on the eighth and sixteenth days of cohabitation, and 1 week after splitting the pair. Blood samples were taken with EDTA from retro-orbital sinus in the same interval (12−15 h) to exclude the influence of hormonal circadian rhythms. A total of 197 samples were collected and centrifuged at 2600 rpm/15 min. After the olfactory test for females the stages of the estrous cycle were determined using microscopy of the vaginal smear. The female was considered receptive at the stage of transition from pro-estrous to estrous, when they attain the greatest readiness to interact with the male (Vasilieva et al., 2017). The concentration of steroid hormones in plasma and urine was determined using ELISA kits (testosterone-ELISA-Best and cortisol-ELISA-Best, Novosibirsk), after conducting a test for the validity of the applied kits. The binding curve of serially diluted aliquots was significantly parallel to the curve of standard solutions (*P*>0.05), which indicated the possibility of using these kits for determining of our analytes. The concentration of all agents in the urine sample was related to the creatinine level assessed using a commercial kit (Creatinine-Agate-Med). The amount of total protein in urine was determined by the method of Bradford (1976). To assess the reaction of a nonspecific link of the immune system, we measured the plasma's peroxidase activity at the endpoint (by Kontsevaya, 2013) using a tetra-methyl-benzidine solution as a substrate.

### Statistical analysis

The statistical data were analyzed with the application of the STATISTICA 6.1 software. Average values were expressed in the text as M±s.e.m. When paired comparison of normally distributed characteristics, the Student's *t*-test was used, abnormally distributed ones used the Mann–Whitney *U*-test. Analysis of variance (Tukey's HSD test) was used for multiple comparisons of traits. Repeated measures analysis of variance (RM-ANOVA) was used to analyze repeated measures.
